# Bio‐Graphene Sensors for Monitoring Moisture Levels in Wood and Ambient Environment

**DOI:** 10.1002/gch2.202200235

**Published:** 2023-02-21

**Authors:** Mohammad Yusuf Mulla, Patrik Isacsson, Illia Dobryden, Valerio Beni, Emma Östmark, Karl Håkansson, Jesper Edberg

**Affiliations:** ^1^ Printed‐, Bio‐ and Organic Electronics RISE Research Institutes of Sweden Bredgatan 35 Norrköping SE‐602 21 Sweden; ^2^ Digital Cellulose Center Bredgatan 35 Norrköping SE‐602 21 Sweden; ^3^ Department of Science and Technology (ITN) Laboratory of Organic Electronics Linköping University Norrköping SE‐601 74 Sweden; ^4^ Ahlstrom Group Innovation Apprieu 38140 France; ^5^ Bioeconomy and Health RISE Research Institutes of Sweden Drottning Kristinas väg 61 Stockholm SE‐114 28 Sweden; ^6^ Stora Enso AB Innovation Centre for Biomaterials Box 70395 Stockholm SE‐107 24 Sweden

**Keywords:** cellulose, humidity sensors, laser‐induced graphene, lignin, moisture sensors, wood

## Abstract

Wood is an inherently hygroscopic material which tends to absorb moisture from its surrounding. Moisture in wood is a determining factor for the quality of wood being employed in construction, since it causes weakening, deformation, rotting, and ultimately leading to failure of the structures resulting in costs to the economy, the environment, and to the safety of residents. Therefore, monitoring moisture in wood during the construction phase and after construction is vital for the future of smart and sustainable buildings. Employing bio‐based materials for the construction of electronics is one way to mitigate the environmental impact of such electronics. Herein, a bio‐graphene sensor for monitoring the moisture inside and around wooden surfaces is fabricated using laser‐induced graphitization of a lignin‐based ink precursor. The bio‐graphene sensors are used to measure humidity in the range of 10% up to 90% at 25 °C. Using laser induced graphitization, conductor resistivity of 18.6 Ω sq^−1^ is obtained for spruce wood and 57.1 Ω sq^−1^ for pine wood. The sensitivity of sensors fabricated on spruce and pine wood is 2.6 and 0.74 MΩ per % RH. Surface morphology and degree of graphitization are investigated using scanning electron microscopy, Raman spectroscopy, and thermogravimetric analysis methods.

## Graphical abstract

A humidity and moisture sensor are produced directly on wood using a bio‐based ink in combination with laser‐induced graphitization. Highly sensitive sensors are achieved which can be used to monitor the moisture content inside of the wood, as well as the surrounding air. The devices are demonstrated in controlled environment using wireless readout electronics.

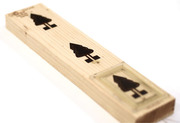

## 1 Introduction

Wood has a long history of being used for construction, with some archaeological findings dating the practice to be more than 10 000 years old. For millennia, wood was the preferred choice as a building material due to its abundance, light weight, and good heat insulation. As cities grew larger, wood was replaced with new materials such as brick and concrete, partly to prevent fires from spreading. However, in modern time, the use of wood in construction has resurfaced due to growing concerns for the environmental impact of materials such as concrete. Wood, being a biobased material, is carbon dioxide (CO_2_) neutral and has been shown to have a lower total CO_2_ equivalence and better recycling possibilities compared to concrete in construction.^[^ ^1^, ^2^, ^3^ ^]^

Although wood has many advantageous properties, it is a hygroscopic material, meaning that it will absorb ambient moisture. High levels of absorbed moisture can lead to growth of mold, with health hazards to people, deformation such as bending, or rotting which can cause structural damage. For this reason, there is a need to monitor both the humidity in the surrounding air and the moisture inside the wood to prevent such degradation at an early stage. Monitoring moisture in wood is important even earlier in the value chain, as timber or boards need to be dried for long time (often 6–24 months) before they can be further processed. The method and kinetics of drying can also have a big impact on the quality of the final product.^[^ ^4^ ^]^ By directly sensing the water content in the timber, this process could be made more efficient. With modern technology, this is becoming reality, as connected internet‐of‐things (IoT) devices can sense various physical and chemical parameters and communicate the information.^[^ ^5^, ^6^ ^]^

IoT devices are exponentially growing in numbers, and they can play vital role in making our society more sustainable and safe. This includes buildings, where IoT can be used to collect important data for making them more energy efficient. It is estimated that the annual cost for repairing water damages in buildings exceeds 500 000 000 € in Sweden alone, which also comes with an environmental cost in terms of energy and material used for the repairs, as well as an impact of the generated waste from the damaged materials.^[^ ^7^ ^]^ While electronics can indeed be used to make both buildings and other structures more sustainable, it is important to make sure that these devices themselves are environmentally friendly and do not contribute to the problem of electronic waste and associated pollution.^[^ ^8^, ^9^, ^10^ ^]^

Making electronics more sustainable, a topic sometimes referred to as green electronics, includes the choice and source of raw materials, manufacturing techniques, and end of life disposal, to name a few factors.^[^ ^11^, ^12^, ^13^ ^]^ Most electronics use minerals mined from the earth as well as synthetic plastics in their construction. Many of these minerals are considered as “critical materials,” and mining is considered an environmentally problematic process.^[^ ^14^, ^15^ ^]^ Also, most plastics are made from fossil materials and can contribute to the problem of microplastics when not disposed appropriately.^[^ ^16^ ^]^ A first step toward greener electronics can therefore involve substituting critical materials with more environmentally friendly alternatives. Researchers are exploring the possibility of using biomass (such as wood) to replace certain materials in electronics, and in recent years, cellulose and lignin have received a lot of attention for this reason.^[^ ^17^, ^18^, ^19^ ^]^ These two biomolecules are the most abundant polymers on the planet and have been shown to have various applications beyond their historical uses. Cellulose fibers, often used to make paper and board, can be converted into nanofibrillar and nanocrystalline cellulose, which both possess remarkable mechanical, chemical, and optical properties.^[^ ^20^, ^21^ ^]^ Nanocellulose has been used in various devices such as batteries, supercapacitors, sensors, and actuators.^[^ ^22^, ^23^, ^24^, ^25^, ^26^, ^27^, ^28^, ^29^, ^30^ ^]^ Lignin is largely a byproduct from the paper industry, and is most commonly burned for energy. However, lignin can also be used to store or generate energy.^[^ ^31^, ^32^, ^33^, ^34^ ^]^ It has also been shown that lignin can be converted into conductive carbon, such as graphite or graphene, through different thermal treatments, such as laser‐induced graphitization (LIG).^[^ ^35^, ^36^, ^37^, ^38^, ^39^, ^40^ ^]^ This conductive carbon can be used to replace metals in certain electronics applications, and by using biopolymers as a precursor, the process can be more environmentally friendly compared to using carbon from mining or fossil sources.^[^ ^41^, ^42^ ^]^

Another important aspect of green electronics is the way we manufacture these components. Traditional electronic circuits are manufactured using energy intense processes and using toxic chemicals; hence, alternative production routes are needed. One manufacturing technique which has shown promise for having a low environmental impact is printed electronics (PE). Using traditional printing methods (such as screen printing) and inks with electroactive particles, PE can produce circuits on various surfaces at low processing temperature, which results in a low energy consumption and an overall lower environmental impact.^[^ ^43^, ^44^ ^]^ PE is not suitable for all electronics applications (e.g., production of microchips) due to the lower patterning resolution (µm resolution) compared to photolithography methods (nm resolution); however, for may many applications (including those described in this work) the resolution of PE is sufficient.

In our recent work, we developed a technique for combining laser‐induced graphitization, printed electronics, and biobased materials, in order to manufacture green electronic circuits and devices.^[^ ^45^ ^]^ The method termed as “lignography,” used a printable ink composed of lignin and cellulose to pattern circuits that were subsequently converted into graphite or graphene upon irradiation of CO_2_ laser beam of 10.6 µm wavelength. Among other examples, we manufactured a humidity sensor using only these forest‐derived components which showed excellent sensitivity over a wide humidity range. However, these sensors were patterned on plastic substrates, which limits their sustainability and eco‐friendliness. In this work, we have applied the lignography technique directly on wood to manufacture sensors that can measure both the ambient humidity as well as the moisture inside the wood. While others have applied LIG on wood for various applications, to the best of our knowledge, this is the first time it has been used for monitoring the properties of the wood itself.^[^ ^36^, ^38^ ^]^ Moreover, the entire process has been carried out in ambient environment without using inert atmosphere of Nitrogen or Argon gases for graphitization. We investigate the kinetics of water transport using the produced sensors and compare the results to commercial sensors. These sensors can lead to more sustainable wooden buildings and other constructions, without relying on critical materials. At the same time, they become easy to handle at end of life, since they do not need to be separated from the wood and can instead follow the usual recycling or destruction flow like other wooden materials.

## 2 Results and Discussion

The traditional method of determining moisture in wood involves a two‐point probe system where conductivity or capacitance is measured between two metallic probes placed over, or inserted into, the top layer of the wood. Such a system is invasive and demands mostly manual measurement, although remote automated measurement systems are available. In this work, we instead fabricated the moisture sensor directly on wood using laser patterning with the goal of achieving more accurate sensor reading and the possibility to remotely monitor the moisture over the entire lifetime of the wood element. At the same time, biobased materials are used to fabricate the electrodes of sensor and also the active sensing material, that is, lignin.

A humidity sensor requires high sensitivity, fast response (as fast or faster than the water transport kinetics inside the wood), low hysteresis, and high stability. Laser graphitization have been used in the past to produce different types of humidity sensors.^[^ ^46^, ^47^, ^48^ ^]^ However, most of these LIG‐sensors were produced by using synthetic polymers (such as polyimide) as the graphitization precursor. Sensors manufactured using bio‐based graphene from wood materials as electrodes and lignin as active sensing material offers the possibility to fabricate simple capacitive or resistive sensors, while at the same time using more sustainable materials compared to synthetic polymers.^[^ ^45^ ^]^ Resistive sensors are widely used since they offer the advantage of ease of fabrication in planar form, good signal linearity and give signals that are relatively easy to read out using simple electronics circuits compared to capacitive sensors.

In initial experiments, four wood types were investigated, namely pine, oak, spruce, and elm. After an initial screening of the printability of lignin ink on the wood surface and the possibility of forming conductive electrodes using laser induced graphitization, it was concluded that pine and spruce showed the most promising results, as cracks were often observed in the carbon on oak and elm.

### 2.1 Fabrication of Sensor on Wood

**Figure 1:**
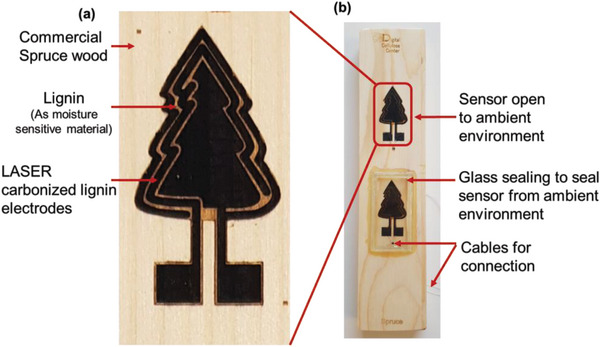
a) Spruce wood coated with lignin ink and graphitized contacts forming interdigitated electrodes (IDE) in the shape of a tree, separated by non‐graphitized lignin acting as moisture sensitive material. b) Two sensors fabricated on a wood surface, one sealed from front side, while the other was kept open to ambient environment.

**Figure** **1**a shows a photograph of a humidity sensor manufactured on commercially available spruce wood. The wood was first coated with a water‐based ink containing lignin and cellulose polymers. The coating was applied using a paint brush over a laser cut stencil mask in the shape of a spruce tree. A CO_2_ laser was subsequently used to produce the two carbon electrodes from the ink coating, while leaving some ink untouched between the electrodes to work as an adsorption layer for moisture sensing. To differentiate between the moisture inside of the wood and the surface moisture adsorbed from the surrounding air, two sets of sensors were used, where one sensor was encapsulated using a glass sealing (Figure 1b).

### 2.2 Optimization of Lignography Parameters

**Figure 2:**
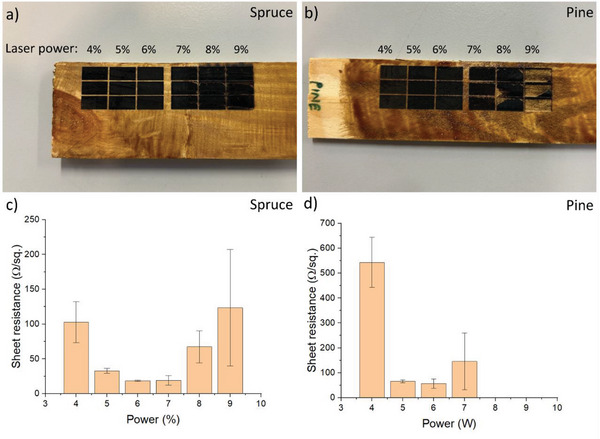
Electrical characterization of graphitized wood. a,b) Photographs of LIG carbon produced on Spruce or Pine at different laser power, corresponding to 4%, 5%, 6%, 7%, 8%, and 9% of the maximum power (30 W). c,d) Sheet resistance values for the squares in (a) and (b).

Before sensor characterization, carbon material obtained from LIG on wood was first investigated. The electrical conductivity of LIG on spruce and pine was measured at different laser powers. The ink was first painted onto the wood, followed by the laser scribing of square patterns. Six different laser power levels were investigated, corresponding to 4%, 5%, 6%, 7%, 8%, and 9% of the maximum power of 30 W. **Figure** **2**a,b shows photographs of the wood pieces after laser graphitization. For the spruce wood, all power settings produced the squares, although small cracks could be seen at levels of 7% and higher. However, for the pine wood, the squares at power of 8–9% resulted in severe cracking such that large pieces fell off. Figure 2c,d shows the corresponding sheet resistance values, measured using a 4‐probe system. The power levels of 8–9% were omitted from the pine sample since they did not form homogenous surfaces.

In our previous work, using the same ink and power settings, but on plastic instead of wood, the sheet resistance was dropping with increasing power.^[^ ^45^ ^]^ Here, instead, we observed the resistance first dropping to a minimum at the power corresponding to 6%, and then again increases. The decreasing resistance was previously explained by an increasing size of graphite crystallites at increasing power. The increasing resistance is likely due to the carbon film cracking at increasing power, which became visually evident at the highest power. The lowest value (18.6 Ω sq^−1^ for spruce) was comparable to the best values obtained in previous work of LIG on wood (8 Ω sq^−1^ by Chyan et al., 10 Ω sq^−1^ by Le et al., 10 Ω sq^−1^ by Ye et al., and 20 Ω sq^−1^ by Dreimol et al.).^[^ ^35^, ^36^, ^37^, ^38^ ^]^ The lowest value of resistance obtained on pine wood was 57.1 Ω sq^−1^. For the remainder of the work, we focused on spruce wood, as we observed more homogenous carbon patterns with fewer cracks compared to pine. This reason behind the difference in homogeneity of graphitized layers between Spruce and Pine could be the fact that Pine wood has inherent resin. This resin might release volatile organic compounds causing localized rupture of surface. Hence, with increasing laser power in case of Pine wood resulted in complete delamination of graphitized layer at 9% laser power. We also used the 6% power as this is where we obtained the optimum sheet resistance in the case of both wood types.

As previously mentioned, one possible reason for the destruction of the graphitized films at higher power is the release of volatile compounds inside the wood which will happen more violently with increasing temperature. This is different from the previous work where the ink was coated on plastic sheets, which contains no volatile compounds.

### 2.3 Scanning Electron Microscopic Topographical and Microstructure Characterization

**Figure 3:**
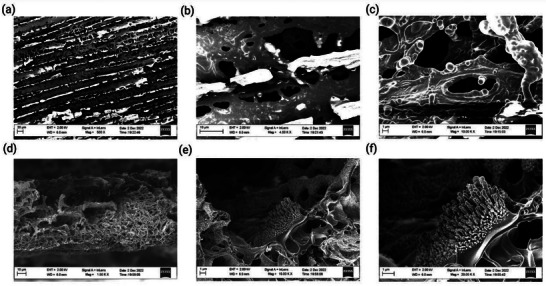
Scanning electron microscopy of flake taken from laser graphitized wood sample. a–c) The top surface with increasing magnification respectively. d–f) The cross section with increasing magnification respectively.

To investigate the microscopic structure of the produced graphitized films, scanning electron microscopy (SEM) was used to investigate the topographical and microscopic structural information. **Figure** **3**a–c shows images taken from the top of a graphitized piece (which was broken off from the wood) at increasing magnification. In Figure 3a, the tracks formed as the laser scans across the surface are observed. Figure 3b,c reveals an open and porous structure of the film. This open structure becomes more apparent when looking at the cross‐section of the piece, as shown in Figure 3d,f at increasing magnification. Figure 3d shows finer pore‐structures at the bottom (the part which was closer to the wood) and larger openings at the top. This can be attributed to the fact that, since more heat is applied closer to the surface, it causes more volumetric expansions when volatiles are released. At higher magnification, we see a variety of structural features, such as bumps and pillars.

### 2.4 Thermogravimetric and Raman Spectroscopy Analysis of Lignin Ink

**Figure 4:**
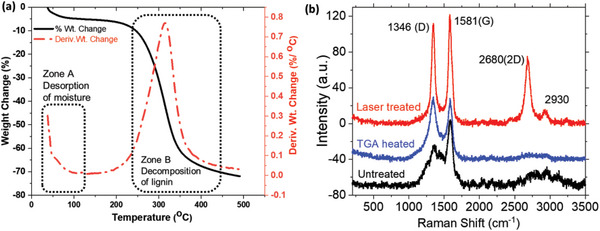
a) Thermogravimetric measurement of lignin ink used in fabrication of the sensor. Percentage weight change (black) and Differential of % weight change (Dotted Red) versus temperature is plotted. b) Raman spectra of untreated lignin ink, lignin ink heated during TGA measurements, and laser treated lignin ink.

The response of the lignin ink to different temperature conditions was investigated using thermogravimetric analysis (TGA) and Raman spectroscopy. First, the low temperature thermal response of the ink was measured by TGA. Dry ink was scraped from the wood and the measurements were performed by varying temperature from 32 to 500 °C while monitoring the change in weight. As observed in **Figure** **4**a, the Zone A of spectrum shows steep decrease in mass in lower range of temperature up to 100 °C, which can be attributed to the residual moisture from the ink being desorbed. At higher temperature, the decrease in mass in Zone B can be attributed to the onset of lignin and cellulose decomposition. Figure 4b shows the Raman spectra of ink that was not heated (untreated), heated to 500 °C during TGA, and laser treated ink. Four distinct peaks can be observed in the graphitized sample, appearing at 1346, 1581, 2680, and 2930 cm^−1^. Three of these peaks can be linked to the characteristic peaks observed in graphene. These are referred to as the D peak (≈1350 cm^−1^), the G peak (≈1580 cm^−1^), and the 2D peak (≈2670 cm^−1^).^[^ ^36^, ^49^ ^]^ The peak at (2930 cm^−1^) could possibly be assigned to the D + D′ peak due to two‐phonon defect‐assisted processes.^[^ ^50^ ^]^ It is observed that the 2D peak is not well‐resolved in the untreated and TGA heated sample, which shows that the high temperature of the laser is needed to convert the lignin into graphene‐like structure. Also, the disorder‐induced D‐band is becoming more narrower and full width half maximum (FWHM) decreases to 56±0.75 cm^−1^ for the laser treated sample. The degree of disorder *I*(D)/*I*(G) is also changing for the laser heated ink from 0.96 (for TGA 500 °C treated sample) to 0.92.

### 2.5 Humidity/Moisture Measurements

**Figure 5:**
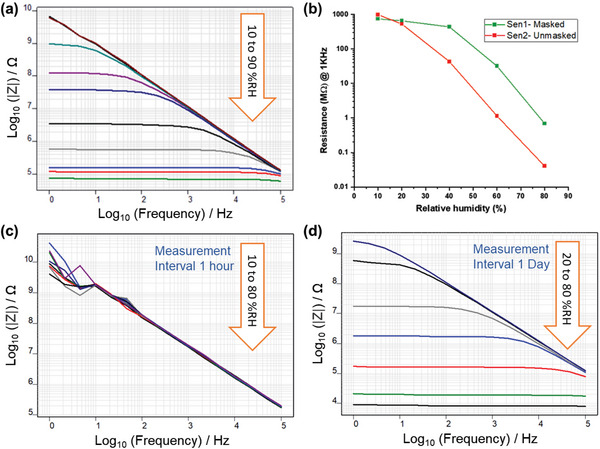
a) Change in impedance of sensor exposed to different humidity levels from 10% to 90%. b) Change in resistance of masked (covered with tape) and unmasked sensors for different humidity levels. (c,d) Change in impedance of sealed sensors when changing the humidity in steps of 10% with 1‐h intervals (c) or 1‐day intervals (d).

The sensors, depicted in Figure 1, were characterized using impedance spectroscopy (1 Hz–100 kHz) in a controlled climate chamber to determine their sensitivity. The relative humidity in the chamber was varied from 10 to 90% RH at 25 °C. First, sensors without any sealing were measured to determine their response to the ambient relative humidity. The wooden blocks with sensor fabricated over its surface was initially dried in oven at 80–90 °C over night and then placed inside the controlled climate chamber held at 10% RH at 25 °C. This was done to exclude as much moisture as possible from the wood so that the measured values would correspond to water that has been adsorbed in the wood from chamber at the different humidity levels. **Figure** **5**a shows the impedance spectra of an unsealed sensor when stepping the humidity from 10% to 90% RH in steps of 10%. The sensors show excellent sensitivity, with the impedance spanning 5 orders of magnitude, which is similar to previously reported humidity sensors.^[^ ^45^ ^]^ After scanning from 10 to 90% RH, the humidity level was brought back to 10% RH to determine the reproducibility of the sensors. The two spectra (straight lines in Figure 5a) almost perfectly overlap with each other confirming negligible hysteresis. To make sure that the change in impedance was only due to the water content in the material between the IDE electrodes (i.e., wood and lignin ink), and not from the carbon electrodes themselves, the change in resistance of the LIG carbon was measured while varying the relative humidity. Figure S2, Supporting Information shows that there is a very small increase in resistance with increasing humidity (opposite to the trend observed in the sensors) and this shift in resistance is negligible compared to the large impedance values of the sensors with interdigitated electrodes with lignin acting as an active moisture sensing material. Figure S4, Supporting Information shows humidity response of sensor fabricated on pine wood, confirming the possibility of fabricating lignographic sensors on different types of wood.

Next, the sensing mechanism of moisture inside the wood was investigated. Two sensors were produced on the same wood block where one was sealed using 3M tape (see Figure S1, Supporting Information), while the other was left exposed to the environment. Figure 5b shows the resulting change in resistance of the two samples when scanned from 10% to 80% RH. While the two sensors had a similar starting value, the change in resistance (real part of the impedance) was smaller for the masked sample compared to the unmasked one. This shows that the water transport kinetics in the wood is slower compared to the surface adsorption directly from the air. However, this setup had its limitations, since the water could be transported laterally in the tape or ink, faster than it is transported in the wood. To ensure that the measured moisture levels corresponded to the actual water content in wood being measured, a new sealing method was employed. This seal, depicted in Figure 1b, used a glass slide attached by a commercially available resin, “Humiseal,” that is widely used in electronics industry to protect electrical components from moisture. With this new setup, any change in the sensor reading must be due to water that has been transported inside the wood, or at least through the top layer of the wood surface. It should be noted that it is the top layer of wood, which is usually measured with the commercial sensors, so the two measurement techniques are comparable in principle, but lignographic sensor offers sensitivity to moisture in wider range, as shown in Figure S5, Supporting Information.

Figure 5c,d shows the impedance spectra of the glass‐sealed sensor when scanning from 10 to 80 or 90% RH. First, each step of 10% RH was done with at least 1‐h intervals. As is evident from Figure 5c, this period was not enough for the wood to come into equilibrium with the new humidity levels. Figure 5d shows the response when an equilibration time of 1 day between steps with increasing humidity was used. Here, the impedance response is clear and resembles the spectra from the open (non‐sealed) sensor in Figure 5a, although the impedance is overall lower for the sealed sample. This is expected if we consider that the open sensor measures the thin layer of water adsorbed on the surface, while the sealed sensor measures the water in the top layer of wood. The “thicker” layer of water in the sealed sample will result in a lower impedance.

To determine if the moisture in the sealed sensors corresponds to the moisture in the top layer of the wood, which may propagate laterally, or the bulk of the wood, an additional sample structure was made. Figure S3, Supporting Information shows this structure, where two sensors were sealed using PET foil and Humiseal resin and furthermore sealed with aluminum foil glued over Humiseal layer. At the bottom of one of the sensors, the wood was milled from back side to make it thinner. Thinning was carried out to speed up the process of penetration of moisture from backside toward the sensor side. This allowed in reducing the waiting period of each step of increasing humidity levels. The impedance response at humidity levels from 10% to 90% RH was measured for the thin (milled) and thick samples. As observed in the Figure S3, Supporting Information, the impedance response for the thin sample (with equilibration time of at least 1 h) shows a clear response to the changing humidity, while the thick wood sample shows no response (similar to Figure 5c). This shows that the moisture is transported through the wood, and that the measured value therefore corresponds to the true bulk moisture content.

The results show that it is possible for the bio‐graphene sensors to track the moisture content both in the ambient air as well as inside the wood. The sensitivity of open lignographic sensor was found to be 2.6 MΩ per % RH for open spruce wood sensor, while that for Pine wood sensor was 0.74 MΩ per % RH, calculated from Figure 5a and Figure S4, Supporting Information, respectively using formula Abs(R10–R90)/Abs(RH10–RH90), where R10 and RH10 are resistance (real part of impedance at 10 Hz frequency) and relative humidity conditions of chamber at 10% RH and R90 and RH90 are those at 90% RH conditions. The sensitivity of sealed sensor was found to be 6.63 MΩ per % RH, considering equilibrium moisture penetrated into the wood at surrounding relative humidity conditions as per Figure 5d. As a comparison, many other thin‐film humidity sensors fabricated using additive manufacturing techniques reports sensitivity in the range of kΩ per % RH for a similar humidity range (180 kΩ per % RH reported by Hassan et al.,^[^ ^51^ ^]^ 442 kΩ per % RH reported by Zhang et al.,^[^ ^52^ ^]^ and 21.7 kΩ per % RH reported by Xia et al.^[^ ^53^ ^]^). These sensors were fabricated on synthetic substrates such as polyethylene terephthalate and using metals and metal oxide materials. More than measuring moisture content, the sensor can be used to track the transport kinetics of water when monitored over time.

Moreover, the lignographic sensors have shown higher sensitivity to moisture levels compared to the commercial moisture level meter as shown in Figure S5 and Table S5.1, Supporting Information. The commercial moisture level meter could not measure the moisture penetrating the wood until exposed to higher (above 70 to 80% RH conditions) while the lignographic sensors have responded to moisture changes within same time frame. Additionally, the pine wood shows lower water permeability, possibly due to denser structure and resin distribution within bulk that inhibits the moisture permeation.

### 2.6 Demonstrators Depicting Applications of Developed Sensors

**Figure 6:**
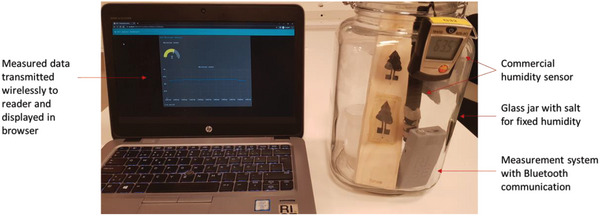
Demonstration of wireless monitoring of moisture in wood. Wood sensor enclosed in glass chamber with saturated NaCl salt solution to maintain humidity ≈64%.

When the validity of the senor signals had been verified, two different sensor readout systems were constructed to monitor the moisture in wood or the surrounding air. The first system, shown in **Figure** **6**, used a microcontroller equipped with Bluetooth that was connected to the sensors. The system was placed in a jar together with a commercial humidity sensor, and the moisture content was controlled by adding sodium chloride saturated salt solution holding the moisture level in chamber around 64%. The microcontroller sent data to a computer by wireless communication, which was displayed in an in‐house developed web app. In this way, the moisture and humidity could be tracked and logged in real time while using the commercial sensor for calibration and verification.

**Figure 7:**
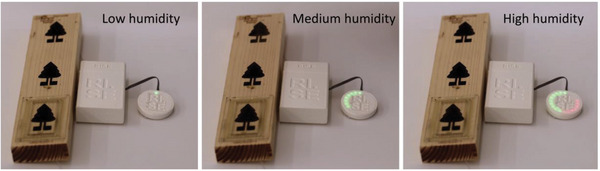
Demonstration of portable sensor readout system with an LED indicator. The LEDs turn on from green to red with increasing humidity level. See Video S1, Supporting Information for the full demonstration.

**Figure** **7** shows another demonstrator version where the moisture levels are depicted using an LED array in circular geometry. More LEDs will turn on as the humidity increases and eventually turning from green to red at higher humidity levels, like heat map graphs. This system can give a more direct readout and does not require any computer. In Videos S1 and S2, Supporting Information, the demonstrators can be seen in action as the humidity is increased by using an atomizer to create a mist. In Video S1, Supporting Information, the mist is blown onto the wood, while in Video S2, Supporting Information, the wood sensor is placed in a sealed plexiglass box depicting conditions in sauna. It should be noted that only the open (non‐sealed) sensors were connected to the sensor readout system in this demonstrator.

## 3 Conclusions

We have demonstrated facile and green method of fabricating moisture sensors directly on the wood using all‐biobased materials such as cellulose and lignin. By employing basic coating techniques and commercially available laser equipment for laser induced graphitization operating in ambient air, highly sensitive (>2 MΩ per % RH) moisture sensors were fabricated on commercial wood blocks without any preprocessing. We demonstrated that the sensors can be used to measure both the surrounding humidity level as well as the moisture content inside the wood. We further demonstrated that the sensors could be connected to a sensor readout system for remotely reading and logging the sensor data. Such systems could help with determining when wood has the ideal dryness during the production of wooden boards, or as an early warning signal to detect moisture damage in buildings which use wood as construction elements. By mitigating the risk for water damage such as mold and rotting, the construction industry may become more inclined to use wooden‐elements in buildings, which is a more sustainable alternative compared to concrete and steel.

## 4 Experimental Section

### Materials

2‐hydroxyethyl cellulose (2‐HEC, MW 90000) DS 1.3, lignosulfonate (lignosulfonic acid sodium salt MW 52000), and boric acid was purchased from Sigma Aldrich., commercial wood samples were purchased from local market “Bauhaus.” The lignin ink was prepared using the procedure developed earlier (lignin–cellulose ratio 1:1).^[^ ^45^ ^]^

### TGA Measurements

Thermogravimetric analysis measurements were carried out using TGA Q500 from TA Instruments. Sample of ≈2 mg was scraped into Aluminum crucible from wood the area coated with lignin ink that was not carbonized by laser during sensor fabrication. Nitrogen flow of 40 mL min^−1^ was maintained. The Ramp method was chosen to obtain the information of temperature effect at in lower temperature range below 100 °C. The ramp of 20 °C min^−1^ from ambient temperature up to 500 °C. Preliminary isothermal step was excluded purposely to obtain weight change information at lower temperatures.

### Raman Spectroscopy

A WITec Alpha 300 RAS system (Ulm, Germany) equipped with a 532 nm excitation laser was used to carry out the Raman spectroscopy measurements. The 50× ZEISS LD EC Epiplan–Neofluar Dic 50×/NA 0.55 objective and an optimized power on the sample of 1 mW to minimize heat induced effects were used in the measurements. All spectra were analyzed using WITec Project Plus 5.1 software. A Lorentzian peak was fitted to obtain D and G band intensities and FWHM. The spectrometer diffraction grating of 600 L mm^−1^ was implemented in the study. The spectra were also corrected for cosmic rays and the background baseline by subtracted a shape‐based correction.

### Sensor Fabrication

Commercially used CO_2_ laser engraver Speedy 300 Laser machine from Trotec served as source of laser for graphitization. The Laser had 10.6 µm wavelength and maximum obtainable power of 30 W. Maximum engraving speed of 3.55 m s^−1^ can be obtained with the machine.

The sensor was fabricated directly on the commercial wooden blocks of Spruce wood sourced from local wood store. The blocks were purchased and cut into ≈15 cm × 4 cm × 1.3 cm sizes. No preprocessing was done on the blocks. Initially a tree shaped pattern was cut on polymer film placed over the wood block acting as a mask. The lignin ink was brush coated over the whole area and left to dry. After drying the laser graphitization was carried out to obtain the interdigitated electrodes in the shape of a tree, signifying the Green all forest‐based sensor for wood. After laser engraving the masking polymer film was removed. A hole was drilled for thin cables, the end of cables was glued to contact pads using conductive carbon paste to hold the cables firmly. In case of sealed sensors, the sensors were either covered with 3M sealing tape or a microscope glass slide was placed over the sensor area with plexiglass frame surrounding the sensor and creating the gap between sensor surface and inner side of the glass. The glass and plexiglass frame both were sealed with overlayer coating of commercial resin “Humiseal.”

### Sensors Electrical Characterization

Impedance spectroscopic measurements were carried out using portable potentiostat “Iviumstat” from Ivium. The frequency scan was recorded from 1 Hz to 100 kHz with AC signal amplitude of 1 V.

The sheet resistance of carbon produced by LIG was measured using a 4‐point probe system from Ossilla (U.K.).

### Sensor Characterization in Controlled Climate Chamber

Sensors were characterized in controlled climate chamber (Make‐vötschtechnik, Model‐LabEvent) where humidity ranges were varied from 10% up to 90% RH at fixed temperature of 25 °C. The sensors were dried for at least 12 h in dry conditions before starting the measurements, to ensure the maximum extraction of moisture from the wood surface and bulk region.

## Conflict of Interest

The authors declare no conflict of interest.

## Author Contributions

All authors contributed to the preparation of this manuscript. V.B., K.H., and J.E. conceived the idea; J.E. developed ink formulation, graphitization for resistivity measurements; Y.M. Fabricated sensors on wood, performed humidity chamber measurements, SEM and TGA characterization, and developed demonstrators along with reading electronics and laptop webapp; I.D. performed Raman spectroscopy and analyzed Raman data; E.Ö. and P.I. helped in providing information about wood types and structures, procurement of wood for sensor fabrication. V.B., E.Ö., K.H., and J.E. and supervised the work.

## Supporting information

Supporting Information

Supplemental Video 1

Supplemental Video 2

## Data Availability

The data that support the findings of this study are available from the corresponding author upon reasonable request.
