# Supplementary material for: Bio‐Graphene Sensors for Monitoring Moisture Levels in Wood and Ambient Environment
Source: Glob Chall. 2023 Feb 21;7(4):2200235. doi: 10.1002/gch2.202200235 (PMC10069311; doi:10.1002/gch2.202200235)
Supplement: Supplementary file 1 — Supporting Information [file GCH2-7-2200235-s001.pdf]

Global Challenges
Open Access

Supporting Information

for *Global Challenges,* DOI: 10.1002/gch2.202200235

Bio-Graphene Sensors for Monitoring Moisture Levels in Wood and Ambient Environment

*Mohammad Yusuf Mulla, Patrik Isacsson, Illia Dobryden, Valerio Beni, Emma Östmark, Karl Håkansson, and Jesper Edberg**

Supplementary information

Bio-graphene sensors for monitoring moisture levels in wood and ambient environment

*Mohammad Yusuf Mulla[1,6], Patrik Isacsson[2,3,6], Illia Dobryde[5], Valerio Beni[1,6], Emma Östmark[3,6], Karl Håkansson[5,6], Jesper Edberg[1,6]**

[1]RISE Research Institutes of Sweden, Printed-, Bio- and Organic Electronics, Bredgatan 35, Norrköping SE-602 21, Sweden.

[2]Linköping University, Department of Science and Technology (ITN), Laboratory of Organic Electronics, Norrköping SE-601 74, Sweden.

[3]Ahlstrom Group Innovation, 38140 Apprieu, France

[4]Stora Enso AB, Innovation Centre for Biomaterials, Box 70395 SE-107 24 Stockholm, Sweden.

[5]RISE Research Institutes of Sweden, Bioeconomy and Health, Drottning Kristinas väg 61, SE-114 28, Stockholm, Sweden.

[6]Digital Cellulose Center, Bredgatan 35, Norrköping SE-602 21, Sweden.

**Corresponding author: jesper.edberg@ri.se*

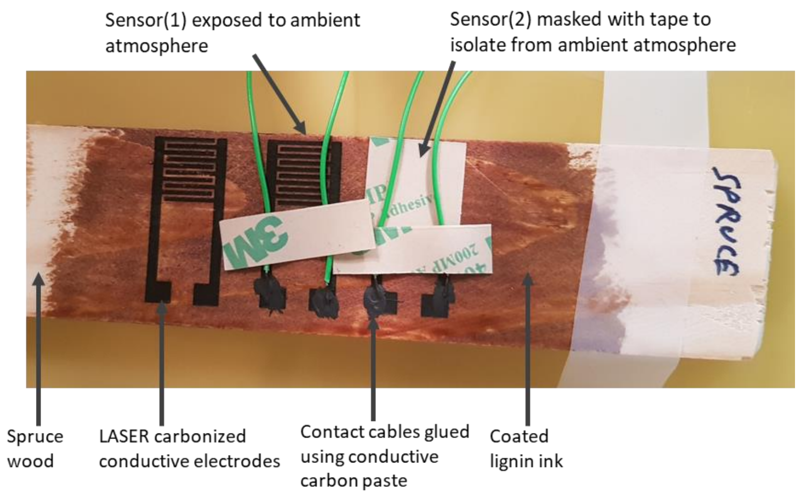

Figure SI1. Spruce wood coated with lignin ink and carbonized contacts forming interdigitated electrodes (IDE) separated by non-graphitized lignin acting as moisture sensitive material. One sensor has been masked by film to prevent interaction of sensor with surrounding moisture, while another sensor has been left unmasked. The measurement was recorded using LCR meter with AC frequency of 1KHz at 1V. It should be noted that the IDE electrode used for these experiments had a different shape compared to the ones in the main manuscript.

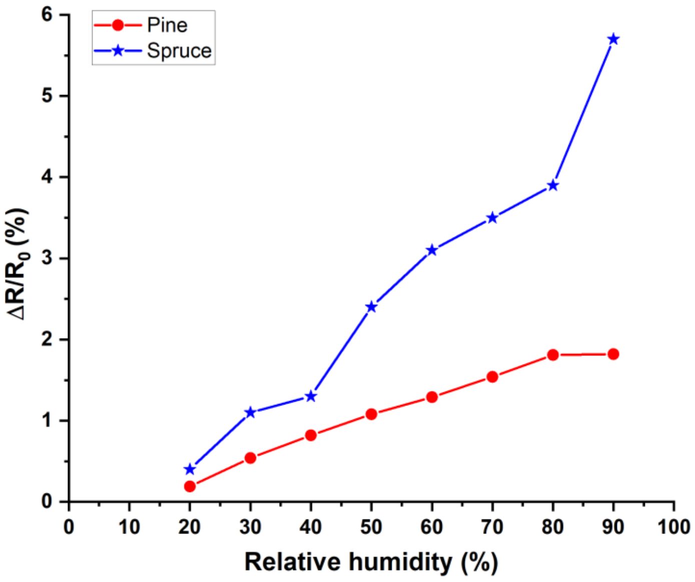

Figure SI2. Change in electrical resistance of carbon produced by LIG on spruce and pine wood when varying the relative humidity. The Y-axis shows the change in resistance (ΔR) divided by the resistance value (R_0) at 10% RH.

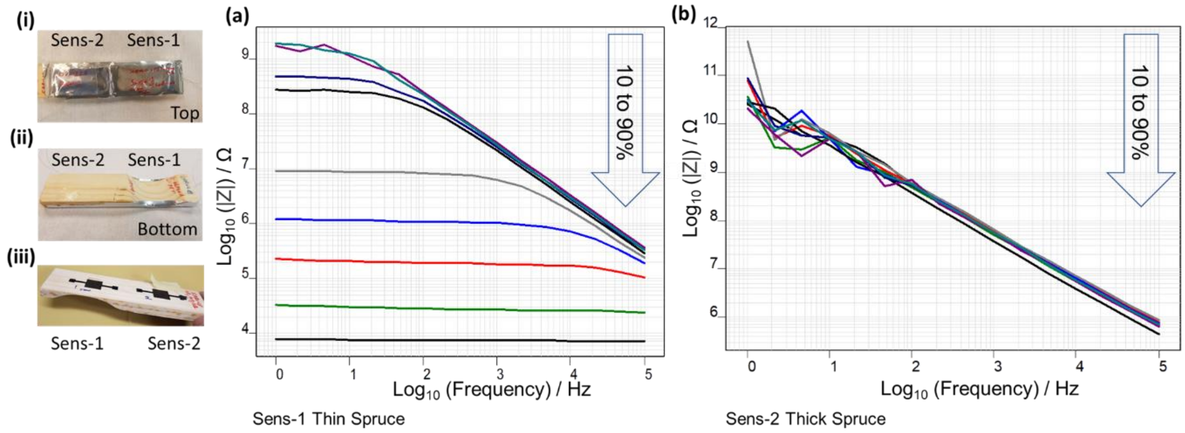

Figure SI3. Spruce wood coated with lignin ink and carbonized contacts forming interdigitated electrodes (IDE) separated by non-graphitized lignin acting as moisture sensitive material. Both sensors were sealed using PET foil and Humiseal resin and furthermore thick aluminum foil was glued over Humiseal layer. At the bottom of one of the sensors, the wood was milled to make it thinner. The impedance response at humidity levels from 10% to 90% RH at 25° C was measured for the thin (milled) and thick samples. It should be noted that the IDE electrode used for these experiments had a different shape compared to the ones in the main manuscript.

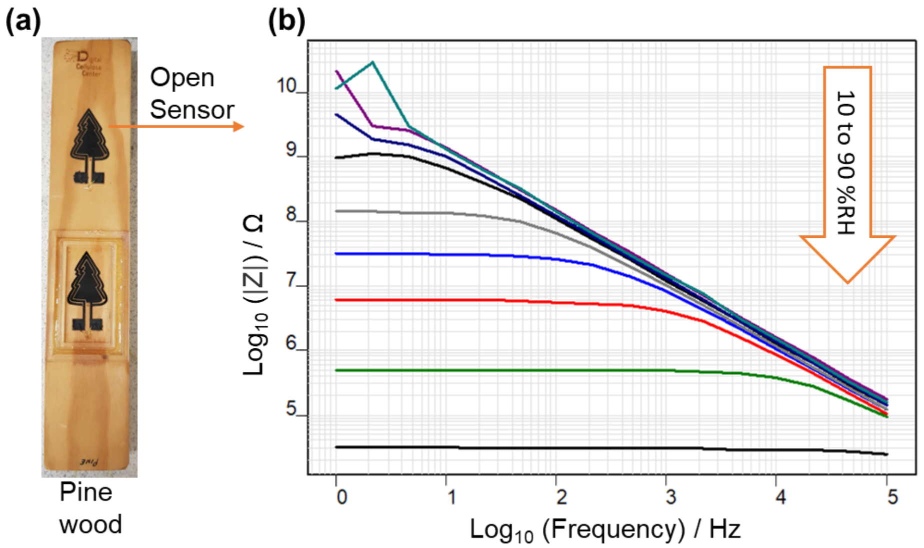

Figure SI4. (a) Pine wood coated with lignin ink and graphitized contacts forming interdigitated electrodes (IDE) in the shape of a tree, separated by non-graphitized lignin acting as moisture sensitive material. (b) Impedance response at humidity levels in the range of 10% up to 90% at 25° C.

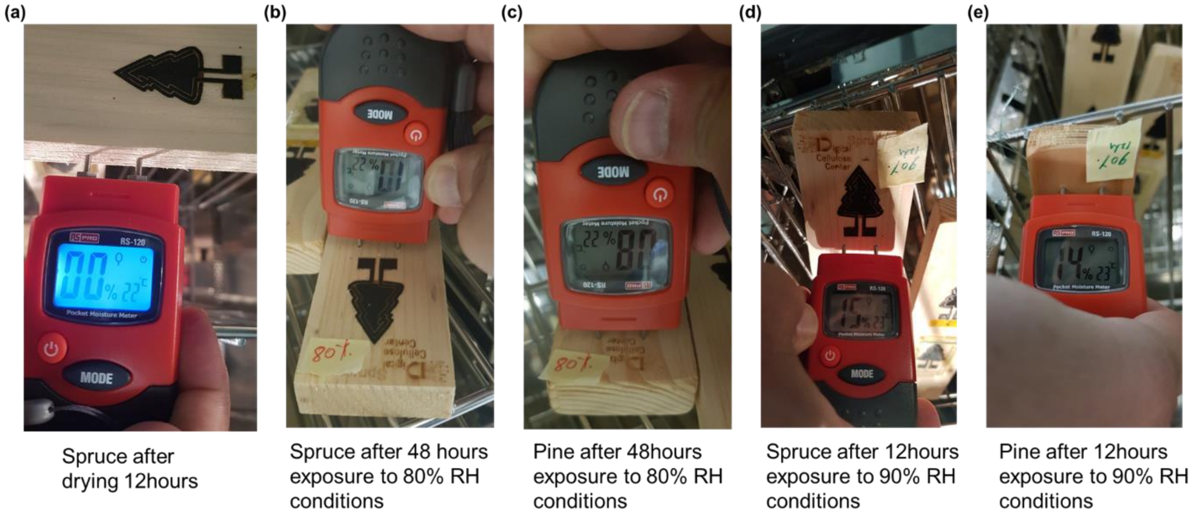

Figure SI5. Moisture inside wood measured using commercial moisture measurement instrument (RS PRO RS-120 Moisture Meter). (a, b, d) and (c, e) are Spruce and Pine wood blocks respectively. Measurements were taken at different exposure periods and moisture conditions in climate chambers. Table SI 5.1 shows the numerical values of recorded moisture levels for respective climate chamber conditions and duration of exposure of wood blocks.

	Climate chamber conditions at fixed temperature of 25 Deg. C. *	Wood type and Measured value using meter	
		Spruce	Pine
1	Drying 10% 12 hours	0 %	0%
2	48 Hours 80% RH	10 %	8%
3	12 Hours 90 % RH	15%	14%

Table SI 5.1 Moisture inside wood measured using commercial moisture meter.

*Note that the meter shows different temperatures other than 25 Deg C, since the chamber was momentarily opened to take measurements using commercial moisture meter, hence ambient temperature is shown in the meter. The chamber is immediately close after these manual measurements and allowed to equilibrate and stabilize at set RH levels at 25 Deg C for measurements with lignographic sensors.

Stability of sensors:-

The sensors fabricated on Spruce wood shown in the supplementary videos have shown responding to moisture over at least nine months after fabrication confirming the stability of the fabricated sensors. Moreover, as shown in Figure SI4, sensor fabricated on Pine wood has been characterized nine months after fabrication. In case of few sensors, it was observed that the carbon paste employed to connect the connecting wires had caused delamination of contact from wooden surface after repetitive measurement cycles in climate chamber. This points to the fact that the graphitized layer is observed to be more inherent part of the wooden surface, while the externally applied carbon paste is

not. This contact detachment issue can be resolved by employing different connecting methods such as clamping, using metal inserts similar to nails or by using metallic conductive glue.
